# Deciphering the Genetic Links between Psychological Stress, Autophagy, and Dermatological Health: Insights from Bioinformatics, Single-Cell Analysis, and Machine Learning in Psoriasis and Anxiety Disorders

**DOI:** 10.3390/ijms25105387

**Published:** 2024-05-15

**Authors:** Xiao-Ling Liu, Long-Sen Chang

**Affiliations:** Institute of Biomedical Sciences, National Sun Yat-Sen University, Kaohsiung 804, Taiwan; d102050006@nsysu.edu.tw

**Keywords:** psoriasis, psychological stress, anxiety disorders, autophagy, machine learning, single-cell RNA sequencing

## Abstract

The relationship between psychological stress, altered skin immunity, and autophagy-related genes (ATGs) is currently unclear. Psoriasis is a chronic skin inflammation of unclear etiology that is characterized by persistence and recurrence. Immune dysregulation and emotional disturbances are recognized as significant risk factors. Emerging clinical evidence suggests a possible connection between anxiety disorders, heightened immune system activation, and altered skin immunity, offering a fresh perspective on the initiation of psoriasis. The aim of this study was to explore the potential shared biological mechanisms underlying the comorbidity of psoriasis and anxiety disorders. Psoriasis and anxiety disorders data were obtained from the GEO database. A list of 3254 ATGs was obtained from the public database. Differentially expressed genes (DEGs) were obtained by taking the intersection of DEGs between psoriasis and anxiety disorder samples and the list of ATGs. Five machine learning algorithms used screening hub genes. The ROC curve was performed to evaluate diagnostic performance. Then, GSEA, immune infiltration analysis, and network analysis were carried out. The Seurat and Monocle algorithms were used to depict T-cell evolution. Cellchat was used to infer the signaling pathway between keratinocytes and immune cells. Four key hub genes were identified as diagnostic genes related to psoriasis autophagy. Enrichment analysis showed that these genes are indeed related to T cells, autophagy, and immune regulation, and have good diagnostic efficacy validated. Using single-cell RNA sequencing analysis, we expanded our understanding of key cellular participants, including inflammatory keratinocytes and their interactions with immune cells. We found that the CASP7 gene is involved in the T-cell development process, and correlated with γδ T cells, warranting further investigation. We found that anxiety disorders are related to increased autophagy regulation, immune dysregulation, and inflammatory response, and are reflected in the onset and exacerbation of skin inflammation. The hub gene is involved in the process of immune signaling and immune regulation. The CASP7 gene, which is related with the development and differentiation of T cells, deserves further study. Potential biomarkers between psoriasis and anxiety disorders were identified, which are expected to aid in the prediction of disease diagnosis and the development of personalized treatments.

## 1. Introduction

For a long time, there have been an extensive number of studies conducted relating to the correlation between psychological stress and physical health. Anxiety is the most common mental health condition, affecting approximately 14% of the population [1,2]. It has attracted widespread attention regarding its association with physical health. Recent studies have shown that psychological stress may be closely related to the occurrence and development of various skin diseases, including autoimmune skin diseases such as psoriasis. Psoriasis is a common chronic inflammatory and recurrent erythematous scaly skin disease. The etiology of psoriasis remains unclear, and it often involves joints and other systemic targets, leading to systemic pathological conditions, with no completely effective therapy currently available.

The prevalence of plaque psoriasis in the population is approximately 0.1% to 3% [3], and it is considered a polygenic hereditary disease resulting from the interaction of genetic and environmental factors, with immune mediation as its main pathogenic mechanism.

Many studies have indicated that psoriasis patients also have a high proportion of symptoms such as anxiety, depression, and insomnia. DSM-5 (Diagnostic and Statistical Manual of Mental Disorders, Fifth Edition) classified psoriasis as a psychophysiological disorder, which manifests various bodily symptoms due to psychological factors [4].

Some studies support the view that emotional stress, usually in the form of stressful events, leads to or exacerbates psoriasis in a higher proportion of cases. Stress may act as a triggering factor for skin disease outbreaks or exacerbations through psychosomatic mechanisms [5]. Although psychological stress has long been considered a factor leading to the onset, aggravation, and recurrence of psoriasis, little is known about the potential physiological mechanisms between the two. Therefore, whether due to illness-induced psychological stress or skin reactions induced by psychological stress, the high proportion of mental illness among psoriasis patients inevitably raises the question of whether there is a comorbid mechanism between mental illness and psoriasis.

The impact of psychological stress on skin health can be realized through various pathways. Psychoneuroimmunology has led to the recognition that the skin is a target of psychological stress, and its response to stress affects the severity and frequency of diseases [6]. Acute stress causes immune cell infiltration into skin tissues, and endogenous stress hormones induce lymphocyte aggregation, leading to overexpression of cytokine genes. Chronic stress inhibits immune system function, resulting in immune imbalance and increased inflammatory responses [7]. In addition to affecting the development and repair of neuronal cells, stress also affects cellular autophagy processes. In recent decades, researchers have begun to realize the potential role of cellular autophagy in the development and progression of psychiatric disorders [8]. Autophagy is an important cellular self-clearing mechanism that maintains cellular health and homeostasis by degrading and recycling damaged or aging proteins and organelles, while also participating in the regulation of cellular oxidative stress and inflammatory responses.

Research has found that patients with acute bipolar affective disorder have decreased expression of the AKT1/mTOR (Alpha serine/threonine-protein kinase/Mammalian target of rapamycin) gene during depressive episodes [9]. NLRP3 (Nucleotide-binding domain, leucine-rich-containing family, pyrin domain-containing-3) plays an important role in regulating IL-1 (Interleukin-1) cytokines and is a major factor in triggering neuroinflammation, leading to subsequent neuropsychiatric disorders. Normal autophagic signaling prevents excessive NLRP3 inflammasome activation and subsequent release of IL-1 family cytokines. Therefore, NLRP3 bridges the relationship between stress exposure and immune activation [10]. Furthermore, certain psychiatric drugs have been shown to affect the autophagy process, thereby improving symptoms of psychiatric disorders. In mouse model studies, mTOR inhibition is associated with abnormal neuronal dendriticization and social interaction defects, while rapamycin inhibition of the mTOR pathway can partially restore abnormal social behavior [11].

These studies suggest that the cellular autophagy process of psychiatric patients may be abnormally affected by gene expression and regulation, leading to abnormal autophagy function, which promotes neuronal aggregation and accumulation of toxic substances, thereby exacerbating the pathological process of psychiatric disorders. Autophagic damage weakens the control of cellular oxidative stress and inflammation, which may be related to the pathogenesis and progression of diseases.

Autophagic dysfunction, in addition to contributing to inflammation, also potentially triggers or exacerbates autoimmune diseases. Psoriasis, in addition to immune cell infiltration into the skin, also promotes excessive proliferation of keratinocytes, overexpression of epithelial fusion molecules, and vascular proliferation. Research by Wang [12] in humans and mice found that deactivating the MAPK (Mitogen-activated protein kinase) signaling pathway reduces autophagy in keratinocytes. The inflammatory cytokines, TNF-alpha (Tumor Necrosis Factor-alpha) and IL-17A (Interleukin-17A), are related to psoriasis and have been pointed out to damage the autophagy function of keratinocytes. Promoting autophagy therapy, such as vitamin D, retinoids, and UVB (Ultraviolet B) phototherapy, has been shown to be effective in psoriasis treatment. Research from various aspects indicates that the pathological mechanism of psoriasis contributes to autophagic dysfunction [13].

Based on these theoretical foundations and previous research, we selected psoriasis and anxiety disorders as the focal points and investigated how psychological stress affects skin health. Anxiety disorders exhibit notably high rates of comorbidity within the realm of mental health, with a majority of individuals affected by anxiety disorders concurrently experiencing other mental or physical illnesses. Moreover, these disorders may serve as precursors to various adult psychiatric conditions, including depression, bipolar disorder, and schizophrenia. In addition to genetic influences, the pathogenesis of anxiety disorders is intricately linked to the emotional stress stemming from life events [14].

It is noteworthy that research indicates a lower proportion of anxiety comorbidity with depression compared to depression and anxiety co-occurrence. This suggests that while a significant number of individuals with depression also experience anxiety, not all individuals with anxiety disorders progress to depression. Studies further demonstrate that anxiety disorders often precede the onset of major depressive disorder (MDD) and can serve as significant predictors of depression development. Consequently, individuals with major depression and comorbid anxiety disorders often encounter greater challenges and complexities in treatment, potentially indicative of a causal relationship between the two conditions [15]. The analysis specifically targeted MDD patients with anxiety disorders.

Additionally, we examined the unique functions and control mechanisms of cellular autophagy in these conditions. It is hoped that a better understanding of the mechanism of this association will provide more effective strategies and directions for future clinical treatment. This will help develop more comprehensive and personalized treatment methods, providing new theoretical and clinical foundations for prevention and treatment.

## 2. Results

### 2.1. Functional Enrichment Analysis

**Figure 1:**
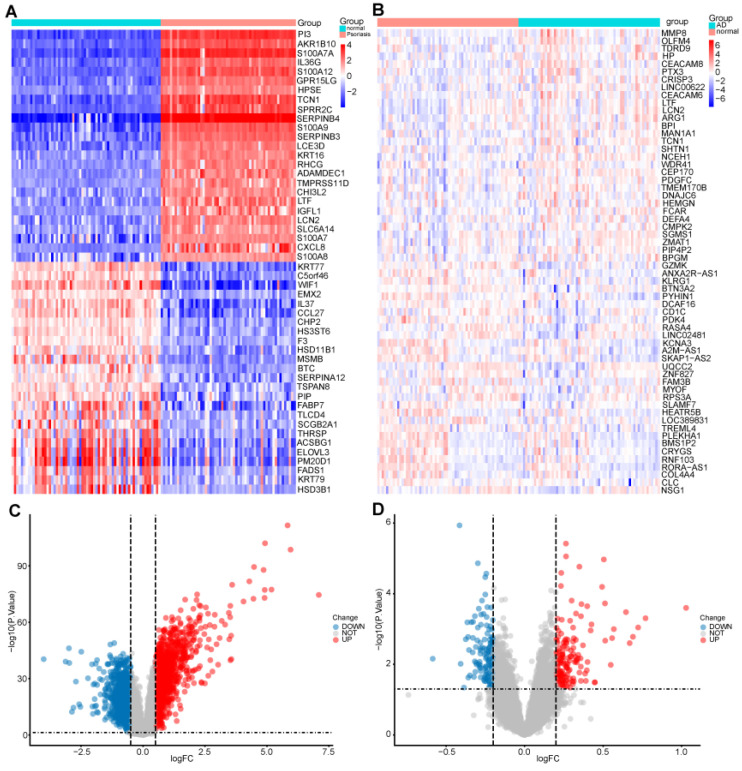
Identification of differentially expressed genes: (**A**) A heatmap of the top 30 DEGs in GSE13355. (**B**) A heatmap of the top 30 DEGs in GSE98793. (**C**) A volcano plot of DEGs in GSE13355. (**D**) A volcano plot of DEGs in GSE98793.

Differentially expressed genes (DEGs) between healthy and diseased groups were identified from two data sets. Generated heatmaps and volcano diagrams were used to visually represent the differentially expressed genes (Figure 1).

**Figure 2:**
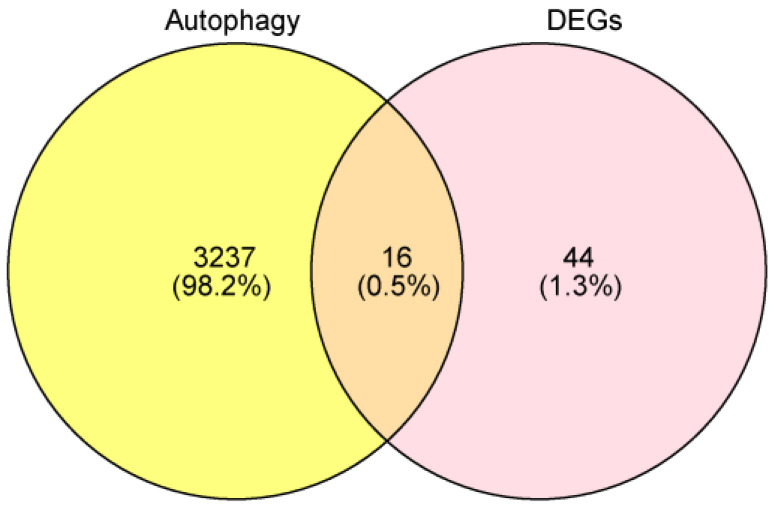
Identification of the shared genes. The Venn diagram shows that 16 genes overlap in psoriasis, anxiety disorder, and autophagy-related genes.

This resulted in a total of 16 autophagy-related DEGs from GSE13355, GSE98793, and autophagy-related genes (ATGs), as shown in Figure 2.

**Figure 3:**
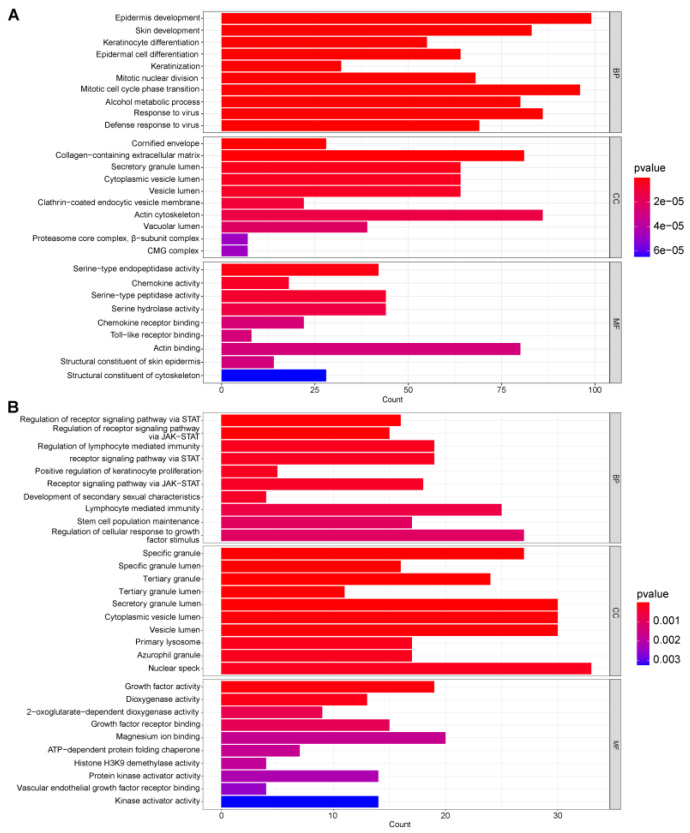
Gene ontology analysis of GSE13355 and GSE98793. GO functional enrichment analysis of the common differentially expressed genes between psoriasis and anxiety disorders: (**A**) The bar graphs of GO enrichment analysis of GSE13355. (**B**) The bar graphs of GO enrichment analysis of GSE98793.

Gene ontology (GO) analysis was conducted to uncover insights into biological processes, cellular components, and molecular functions. The results are summarized in Figure 3A,B and highlight the top 10 GO items.

**Figure 4:**
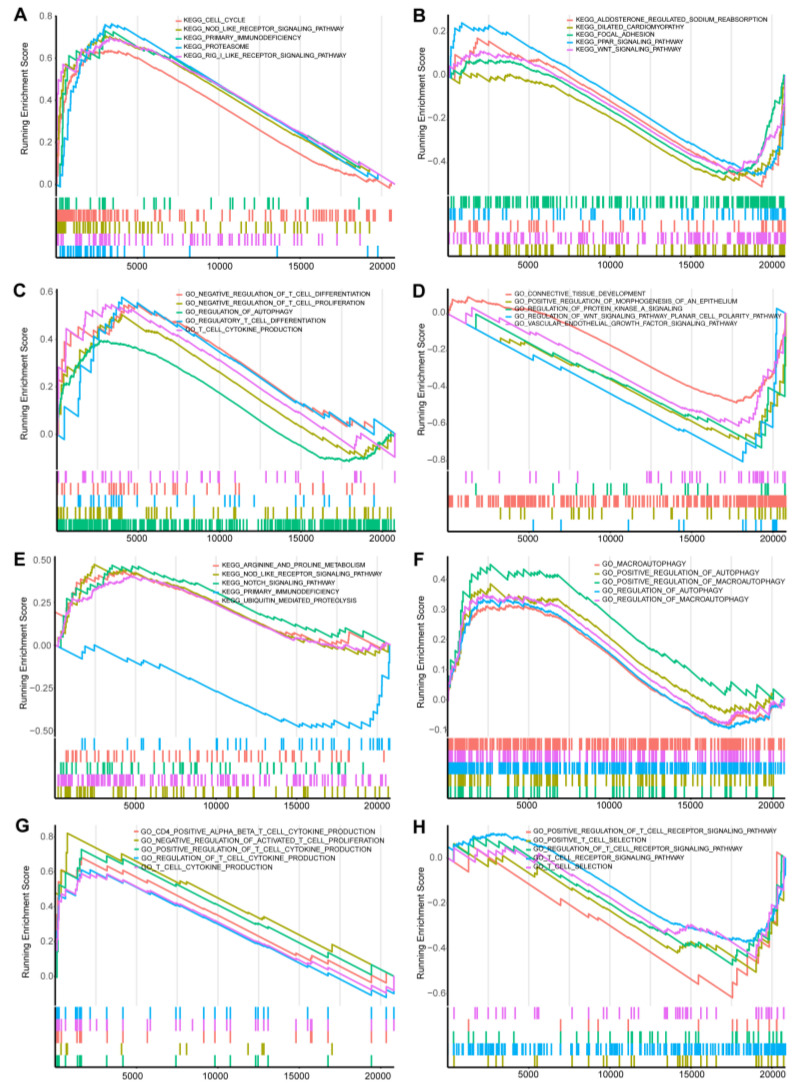
GSEA (Gene Set Enrichment Analysis) analysis of GSE13355 and GSE98793: (**A**) Enriched KEGG pathway with high expression of GSE13355. (**B**) Enriched KEGG pathway with negative expression of GSE13355. (**C**) Enriched bioprocess with high expression of GSE13355. (**D**) Enriched bioprocess with negative expression of GSE13355. (**E**) Enriched KEGG pathway with high expression of GSE98793. (**F**) Enrichment of biological process related to autophagy expression in GSE98793. (**G**) Enrichment of biological processes related to high immune expression in GSE98793. (**H**) Enrichment of biological processes related to negative immune expression in GSE98793.

In the GSE13355 data set, the DEGs identified exhibit significant enrichment in the regulation of diverse cellular processes, encompassing autophagy, T-cell migration, proliferation, cytotoxicity, and communication. This set of genes exerts a negative influence on biological processes associated with T-cell maturation and differentiation (Figure 4C), and it is also related to the regulation of the WNT signaling pathway, vascular–endothelial growth factor signaling pathway, protein kinase A signaling, connective tissue development, and regulation of the morphogenesis of epithelium (Figure 4D). Moreover, it plays a role in activating pathways related to immune recognition, while concurrently inhibiting the WNT signaling pathway and PPAR (peroxisome proliferator-activated receptor) signaling pathway, as shown in Figure 4A,B.

In the case of the GSE98793 DEGs, they are predominantly enriched in the regulation of macroautophagy, autophagy, and mitochondrial autophagy (Figure 4F). This gene set contributes to the activation of T-cell cytokine production while suppressing T-cell maturation and differentiation. Additionally, it negatively regulates biological processes related to cell communication (Figure 4G,H). Kyoto Encyclopedia of Genes and Genomes (KEGG) analysis reveals that these DEGs are involved in activating pathways associated with immune recognition and pathways related to arginine and meglumine metabolism. Simultaneously, they inhibit antigen presentation, potentially leading to primary immune deficiencies, as shown in Figure 4E.

### 2.2. Hub Gene Identification

**Figure 5:**
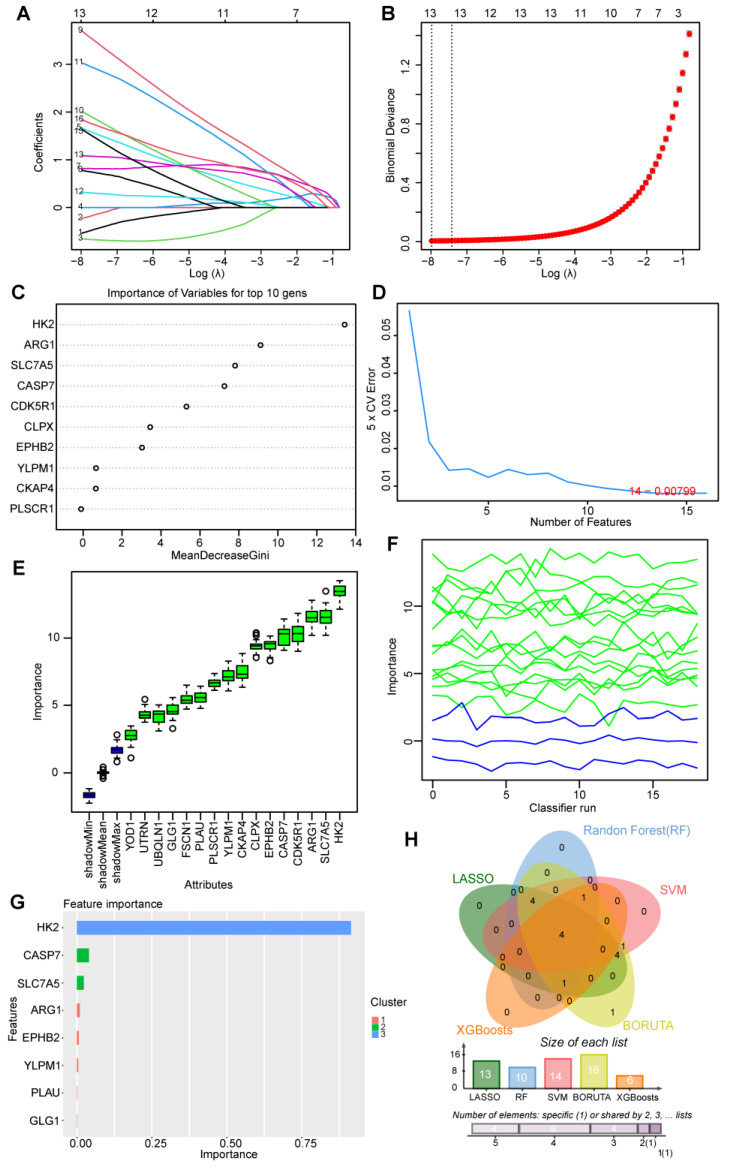
The screening of candidate diagnostic genes using five machine learning algorithms: (**A**) Coefficient RIF profile plot of the LASSO model showed the selection of the optimal parameter l (lambda). (**B**) Tenfold cross-validation to select the optimal tuning parameter log (lambda) in the GSE13355 data set. (**C**) The top 10 genes according to their discriminant ability in the RF algorithm. (**D**) Shared diagnostic genes were selected by using the SVM-RFE algorithm. (**E**,**F**) Shared diagnostic genes were selected by using the BORUTA algorithm. (**G**) Six diagnostic genes were selected by using the XGBoosts algorithm. (**H**) Venn diagram showing the optimal diagnostic biomarkers.

The 16 candidate genes were submitted into five machine learning algorithms—Xgboosts (eXtreme Gradient Boosting), BORUTA, SVM-RFE (Support Vector Machine Recursive Feature Elimination), RF (Random Forest), and LASSO (Least Absolute Shrinkage and Selection Operator), and the results are as follows:

- The LASSO regression algorithm was applied to identify 13 potential candidate genes out of 16 candidate genes. (Figure 5A,B)
- The RF machine learning algorithm was also carried out to rank the 16 candidate genes in light of the variable importance of each gene, and the top 10 important genes with the MeanDecreaseGini score ranking were extracted. (Figure 5C)
- The SVM algorithm identified 14 genes with the lowest 5×CV error. (Figure 5D)
- We used the R package “Boruta”, and “mlbench” to run the BORUTA algorithm to rank the importance of the 16 candidate genes. The result showed that the whole 16 genes were confirmed to be retained without deduction. (Figure 5E,F)
- Then, we inputted the above 16 genes into the XGboosts algorithm classifier, and six genes were selected. (Figure 5G)

We determined which genes overlapped from the five machine learning algorithms, and obtained four hub genes: ARG1, CASP7, SLC7A5, and YLPM1 (Figure 5H).

### 2.3. Diagnostic Model Construction

**Figure 6:**
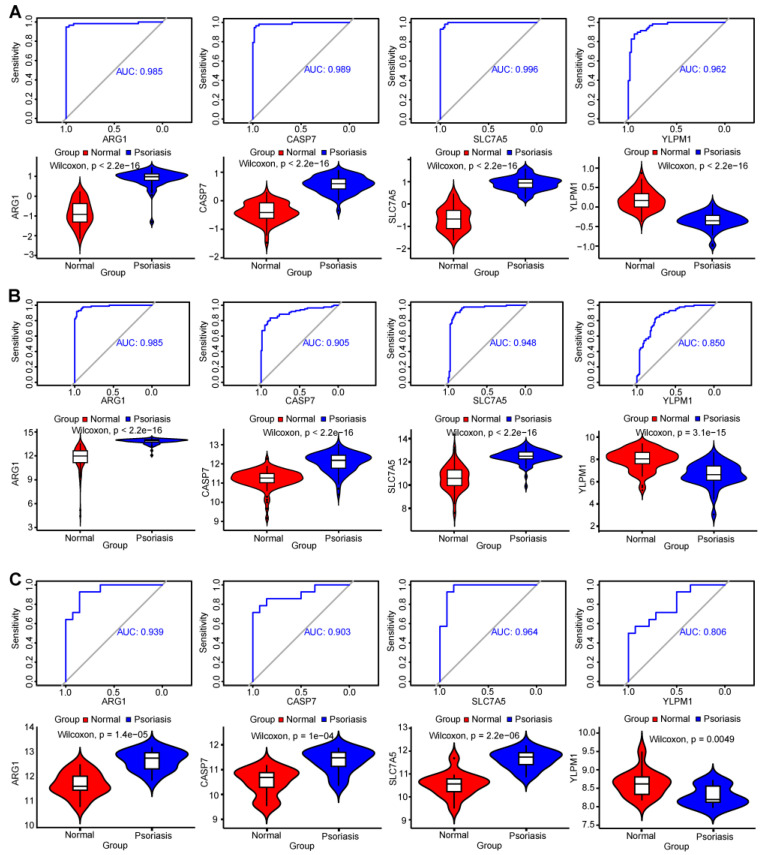
Exploration of the diagnostic value of the 4 shared genes: (**A**) ROC curves showing the diagnostic value of 4 shared genes in the GSE13355 as a training data set. Violin plot showing the gene expression level for the 4 shared genes in the training data set. (**B**) ROC curves showing the diagnostic value of 4 shared genes verified by the GSE30099. Violin plot showing the gene expression level for the 4 shared genes in the validation data set. (**C**) ROC curves showing the diagnostic value of 4 shared genes verified by the GSE34248 data set. Violin plot showing the gene expression level for the 4 shared genes in the validation data set.

The ROC (receiver operating characteristic) curve results showed that all four genes had high predictive ability in the training data set (GSE13355) (Figure 6A). Meanwhile, we performed validation in the other two data sets and acquired a similar result (Figure 6B,C).

The four key genes gained relatively high predictive value in the training data set GSE13355. The AUC (area under the curve metric) values were used to evaluate each diagnostic gene.

Simultaneously, the expression levels were found of the four signature genes in the GSE13355 data set using a violin plot (Figure 6A). The plot clearly illustrates elevated gene expression of the hub genes in the psoriasis group, with the exception of the YLPM1 gene.

### 2.4. The Hub Genes and Immunocyte Infiltration in Different Disease Subtype Patterns

**Figure 7:**
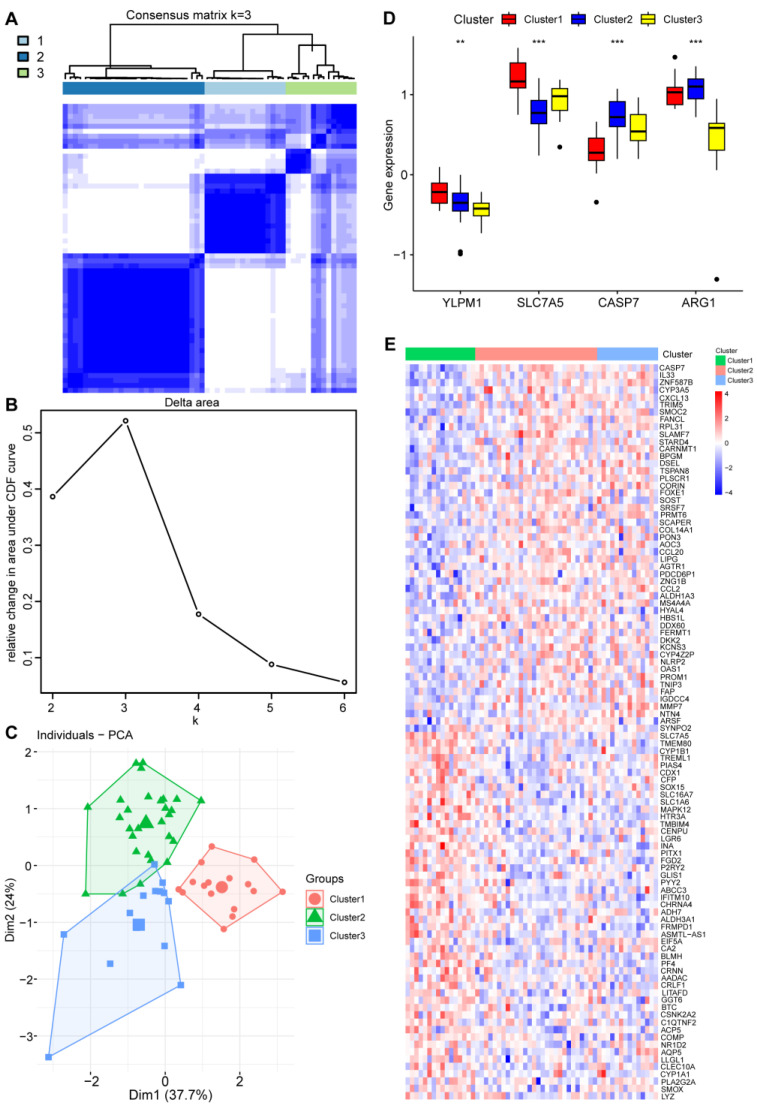
Identification of the molecular subtype clusters based on the 4 shared genes: (**A**) Consensus clustering matrix when k is 3. (**B**) Representative CDF delta area curve. (**C**) Visualization of the distribution of the 3 clusters by principal component analysis. (**D**) The box diagram showing the 4 hub genes expression in the three disease subtypes of psoriasis. The Kruskal test was used. (**E**) A heatmap of the top 30 DEGs for the three disease subtypes. *** *p* < 0.001; ** *p* < 0.01.

Utilizing the hub genes to perform unsupervised clustering on the psoriasis gene data set using the “ConsensusClusterPlus” method. The optimal number of subtypes (denoted as k = 3) was determined by examining key metrics including CDF (cumulative distribution function) curves, consensus scores, and consensus matrices (Figure 7A,B).

These analyses help define distinct subtypes within the psoriasis patient population. Three distinct disease subtypes were distinguished. Principal component analysis (PCA) manifested that psoriasis samples can be further categorized into three groups (Figure 7C). The box diagram shows the four hub genes expression in the three disease subtypes of psoriasis, and a heatmap of the top 30 DEGs for the three disease subtypes, as shown in Figure 7D and Figure 7E, respectively.

### 2.5. Biological Properties of Different Disease Subtype Patterns

**Figure 8:**
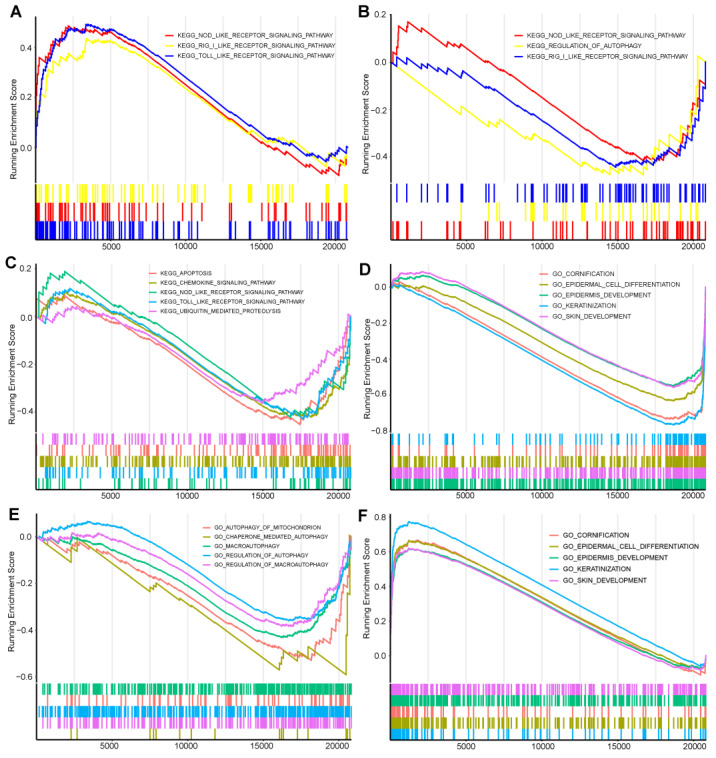
GSEA analysis of subtypes of disease: (**A**) KEGG pathway of cluster 1, the enrichment KEGG pathway with high expression. (**B**) KEGG pathway of cluster 2, the enrichment KEGG pathway with high expression. (**C**) KEGG pathway of cluster 3, the enrichment KEGG pathway with high expression. (**D**) GO enrichment of cluster 1. (**E**) GO enrichment of cluster 2. (**F**) GO enrichment of cluster 3.

We compared the three disease subtypes to understand biological properties, and the KEGG pathways and biological processes were compared. The results indicated that disease subtype 1 activates immune recognition pathways, cellular autophagy, and T-cell migration, while inhibiting normal skin development and keratinization (Figure 8A,D).

Disease subtype 2 participates in the activation of T-cell activity, inhibits immune recognition pathways and cellular autophagy, suppresses Th17 (T helper 17) cell function, induces autoimmunity, and inhibits normal skin development and keratinization (Figure 8B,E).

Disease subtype 3 is highly expressed in normal skin development and keratinization. It inhibits immune recognition pathways, apoptosis, and T-cell migration (Figure 8C,F).

These results provide detailed insights into the gene expression patterns and enriched pathways for each subtype of the disease, shedding light on the underlying biological mechanisms and potential therapeutic targets.

### 2.6. Immunocyte Infiltration of the Disease Subtype

**Figure 9:**
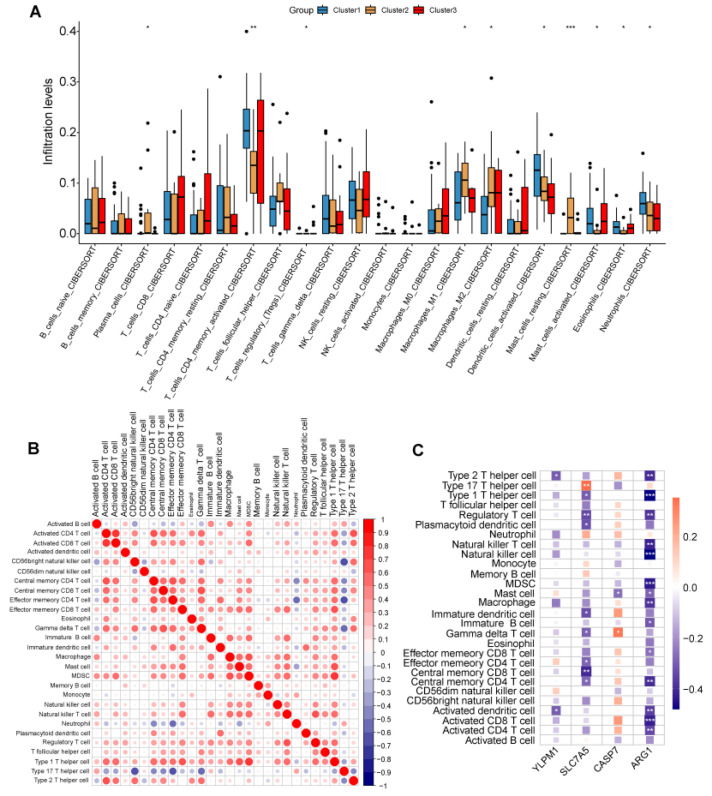
Immune cell distribution of disease subtypes: (**A**) Boxplots showing differences in immune infiltration between the three disease subtypes. (**B**) Correlation matrix of the immunocyte proportions in all samples. Red and blue colors respectively represent positive and negative correlations. (**C**) Correlation analysis between hub genes and immune cells. *** *p* < 0.001; ** *p* < 0.01; * *p* < 0.05.

Cibersortx identified immune cell infiltration patterns and distinct profiles across three disease subtypes. In disease subtype 1, there was elevated expression of DC cells (Dendritic cells), eosinophils, and neutrophils. Disease subtype 2 exhibited increased expression of plasma cells, macrophages, and resting mast cells. Disease subtype 3 demonstrated heightened expression of CD4 (Clusters of differentiation 4) memory-activated T cells and activated mast cells. Interestingly, regulatory T cells (Tregs) consistently displayed significant downregulation across all three disease subtypes. These variations may play pivotal roles in immune system modulation and contribute to the underlying mechanisms of these diseases (Figure 9A).

Moreover, the relationship between infiltrating immune cells and the hub genes expression was evaluated (Figure 9B). The results revealed a noticeable negative correlation between most immune cells and hub genes. Specifically, the SLC7A5 gene showed a positive correlation with Th17, while the CASP7 gene exhibited a positive correlation with gamma delta T cells, as depicted in Figure 9C. These findings underscore the potential significance of inflammatory components in the development of psoriasis and suggest novel regulatory roles of hub genes in immune function.

### 2.7. Transcription Factor and Hub Gene Interactions

**Figure 10:**
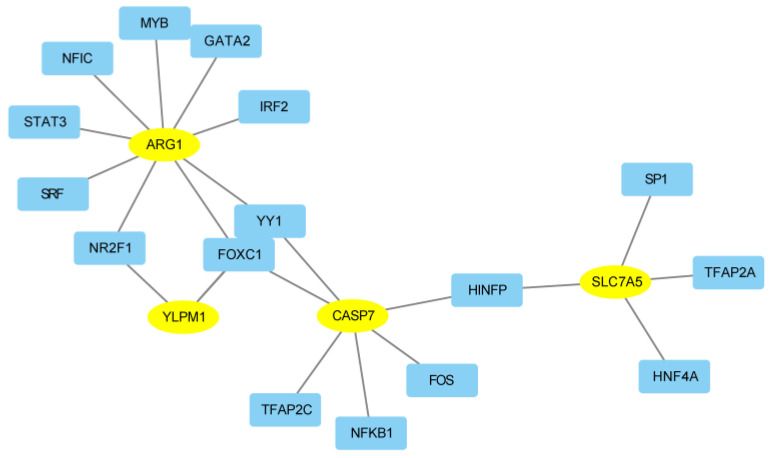
The gene–TF regulatory networks of the 4 genes.

A total of 16 TFs (transcription factors) were obtained, which regulate the hub genes. The TFs–Hub genes network was constructed, including 20 nodes and 21 edges. The resulting TF–gene interaction network is depicted in Figure 10. Specifically, ARG1 is modulated by nine TFs, CASP7 is influenced by six TFs, SLC7A5 is under the control of four TFs, and YLPM1 is regulated by two TFs.

### 2.8. Single-Cell Analysis Reveals Cell Subtypes

To illustrate the characteristics and changes in gene expression on skin lesion cells, we obtained the single-cell RNA sequencing data of GSE151177 from the GEO (Gene Expression Omnibus) database, which included 24,354 cells from 13 human psoriasis skin and 5 healthy volunteer skin, and utilized the Seurat R software package (version 5.0.1) within the R environment (version 4.3.0.1) for downstream single-cell RNA sequencing data analysis, which involved subcluster analysis and visualization using the t-distributed stochastic neighbor embedding (t-SNE), and Uniform Manifold Approximation and Projection (UMAP) approach. The skin cells derived from both psoriatic and normal tissues were categorized into 25 distinct clusters. These clusters were primarily comprised of five cell types, namely, keratinocyte, dendritic cells, monocyte/macrophage, T cell/natural killer cells, and melanocyte cells, as revealed by the unique gene signatures associated with each subcluster.

**Figure 11:**
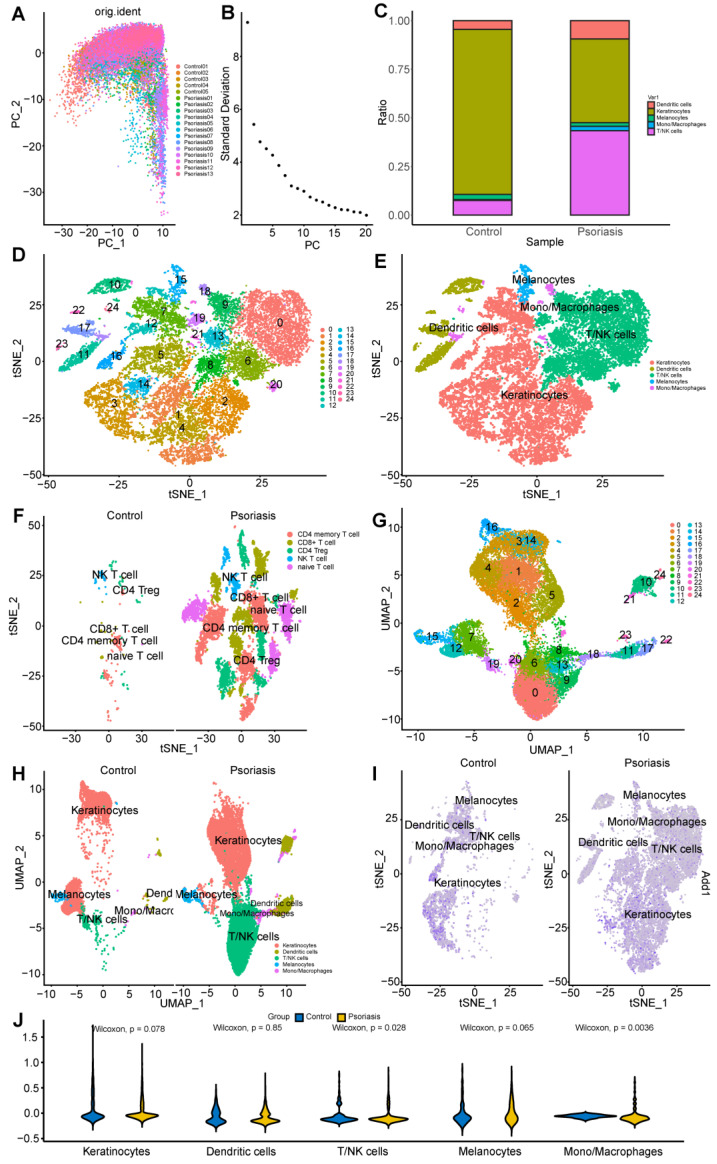
(**A**,**B**) To assess the quality of the scRNA-seq (single-cell RNA sequencing) data, visually assess the presence of batch effects by PCA. (**C**) Stacked bar plot to demonstrate cell percentage between control and psoriasis samples. (**D**) tSEN visualization showing the identified 25 cell clusters; (**E**) Identified Keratinocyte, Dendritic cells, T/NK (Natural killer T cells) cells, Melanocyte, and Monocyte/Macrophages. (**F**) The differences in cell components between the two groups. (**G**) UMAP visualization showing the identified 25 cell clusters. (**H**) Depicting cell clusters, and the differences in cell components between two groups. (**I**) The module score between control and psoriasis samples. (**J**) Violin plot demonstrates the statistical significance of the five cells in two groups.

A significant and noteworthy finding was the statistically significant difference in the abundance of T cells within psoriasis tissue (Figure 11C). As a result, our investigation extended further into the exploration of the trajectory of these key hub genes in T cells at the single-cell level.

### 2.9. T-Cell Subtype Analysis

**Figure 12:**
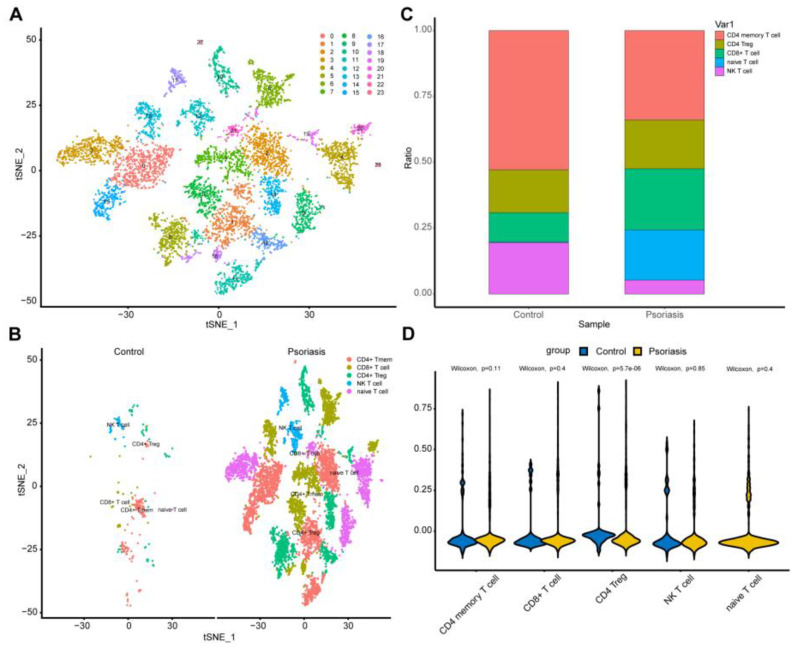
T-cell subtype analysis: (**A**) tSEN visualization showing the identified 24 cell clusters; (**B**) Identified CD4+ T memory cell (CD4+ Tmen), CD8+ T cell, CD4+ T regulatory cell (CD4+ Treg), NK cell, and naive T cell; (**C**) Stacked bar plot to demonstrate cell percentage between control and psoriasis samples. (**D**) Violin plot demonstrates the statistical significance of the five cells in two groups.

T-cell subtype analysis went through further steps of dimensionality reduction clustering, batch effect removal, and the annotation of T-cell subtypes, resulting in the identification of NK cells, Tregs, CD4+ T memory cells, CD8+ T cells (cytotoxic T lymphocytes), and naive T cells. The result of a cellular proportion examination revealed that NK cells and CD4+ T memory cells were remarkably decreased, while CD8+ T cells and naive T cells were substantially increased. (Figure 12C,D). The visualization technique, t-SNE, effectively showcased the differences in T-cell composition, emphasizing significant cellular variances between the disease and normal groups (Figure 12A,B).

### 2.10. Pseudotime and Trajectory Analysis

In the context of single-cell pseudotime analysis, our investigation centered on the developmental trajectory of T cells within the disease group, specifically exploring a distinct T-cell subpopulation. The heatmap representing cellular development disclosed the following trends:

- The CASP7 gene exhibited high expression in the initial phases of T-cell development, followed by a gradual decrease over time.
- The SLC7A5 gene displayed elevated expression in the early phases of T-cell development, followed by a decline, but interestingly, it resurged with significant expression in the later stages of cellular development.
- The ARG1 and YLPM1 genes showcased prominent expression in the advanced stages of T-cell development.

**Figure 13:**
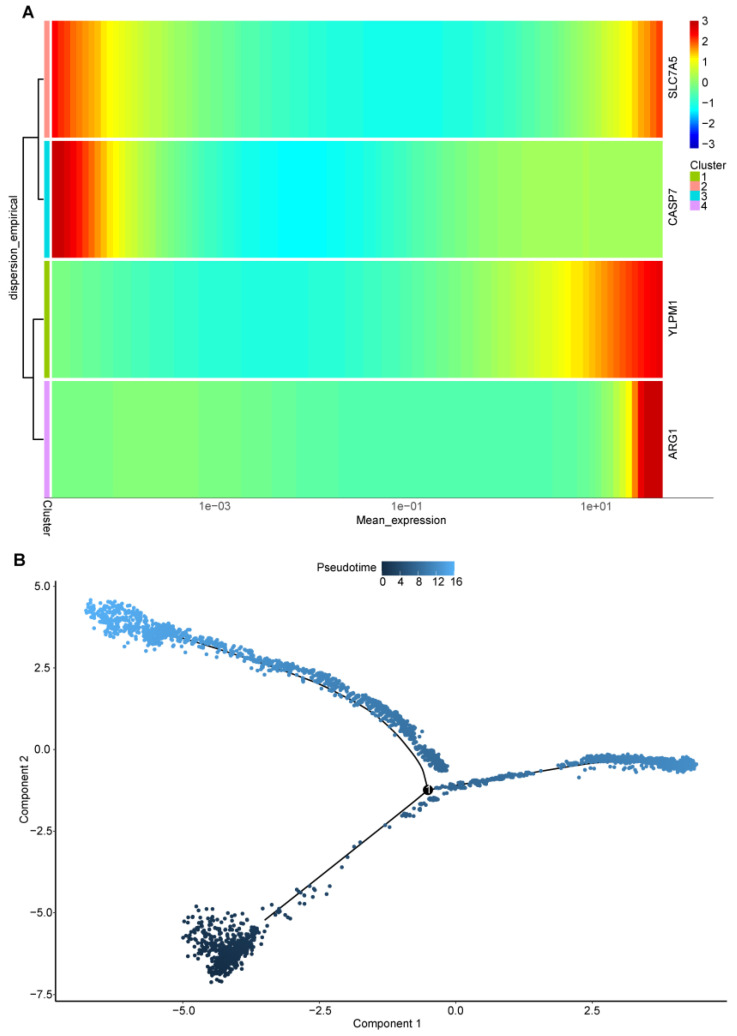
Pseudotime and trajectory analysis: (**A**) Heatmap of the four hub genes, expression along the pseudotime trajectory using Monocle. (**B**) Distinct states of cells identified by pseudotime analysis. Focused Monocle trajectory analysis including only the T-cell cluster.

The pseudotime graph visually delineated the trajectory of cells, portraying an initial stage in deep blue that underwent a branching event, leading to two distinct cell lineages, with cells in the later developmental stage depicted in light blue (Figure 13B).

Transcriptome analysis underscored that the CASP7 gene was notably upregulated in the disease group. Further examination in cellular expression analysis revealed significant overexpression specifically in DC cells. Single-cell gene localization accentuated its elevated expression in both the disease group and DC cells. Pseudotime analysis unraveled the temporal dynamics, with the CASP7 gene showing heightened expression at the beginning of T-cell development followed by a gradual reduction over time (Figure 13A).

### 2.11. Cell Communication Analysis

For cell communication analysis within the disease group, the expression levels of the four hub genes were utilized to generate scores through the AddModuleScore function.

**Figure 14:**
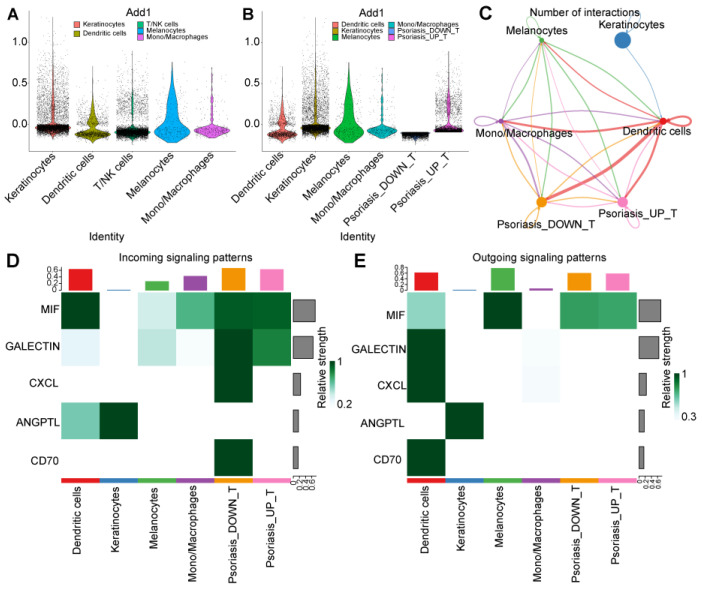
The results of cell–cell communication analysis: CellChat was used to infer cell–cell interactions using psoriasis scRNA seq data (GSE151177): (**A**) Violin plot of cell cluster module score, (**B**) dividing T-cell cluster into high and low module score groups to evaluate cell–cell communication. (**C**) Networks of cell–cell inferred interactions between each cell type. The width of the edge indicates the strength of interactions between cell types. (**D**,**E**) Heatmap visualizing the possible incoming or outgoing signaling pathways among the keratinocyte, melanocyte, and immune cells.

**Figure 15:**
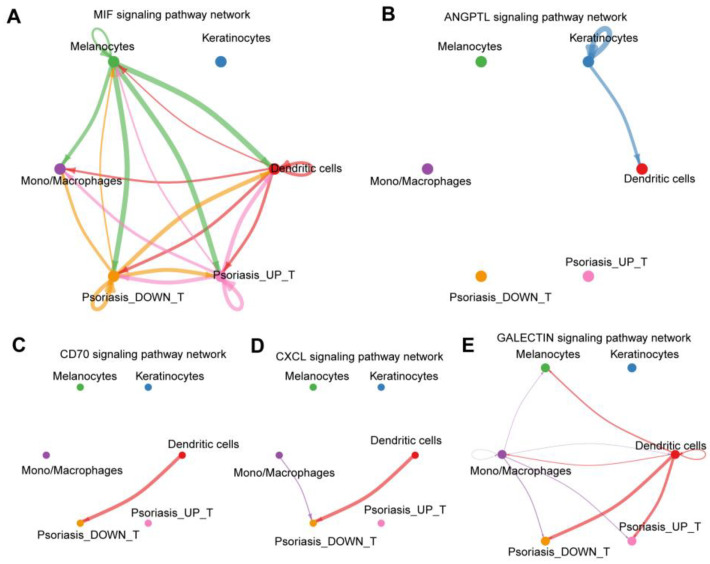
Global intercellular communication among five cell types. The line width represents the intensity, and the arrows indicate direction communication: (**A**) MIF signaling pathway network. (**B**) ANGPL signaling pathway network. (**C**) CD70 signaling pathway network. (**D**) CXCL signaling pathway network. (**E**) GALECTIN signaling pathway network.

The outcomes revealed elevated hub gene expression in keratinocytes, DC cells, and T/NK cells. Further exploration involved the extraction of T/NK cells, leading to the formation of two groups based on median expression levels (“Psoriasis_UP_T” and “Psoriasis_DOWN_T”) for subsequent cell communication analysis (Figure 14A,B). The findings unveiled that keratinocytes emitted ANGPTL (angiopoietin-like) signals to DC cells. DC cells reciprocated by dispatching CD70 (cluster of differentiation 70), CXCL (CXC motif chemokine ligand), GALECTIN, and MIF (Macrophage migration inhibitory factor) signaling pathway signals. T cells in the “Psoriasis_UP_T” group exclusively received MIF/GALECTIN signaling pathway signals, while T cells in the “Psoriasis_DOWN_T” group received a more diverse set of signals, encompassing CD70, CXCL, GALECTIN, and MIF signaling pathways (Figure 14C–E and Figure 15).

## 3. Discussion

A lot of research in the past has focused on the relationship between anxiety, depression, and psoriasis, but the correlation has been controversial. Some studies suggest that patients with early-onset psoriasis (EOP) are more susceptible to anxiety and depression [16]. However, some studies have pointed out that late-onset psoriasis (LOP) is indeed significantly related to anxiety and depression, but no significant correlation was observed for EOP. Chronic inflammatory complications are common in patients with LOP, such as type 2 diabetes, obesity, and autoimmune thyroiditis. Some scholars also believe that lop is a subtype of psoriasis. However, some studies suggest that patients with EOP are more susceptible to anxiety and depression [16]. The above-mentioned positive and negative research results all mean that the relationship between psoriasis and mental stress still needs to be further explored. While these disorders are considered psychosomatic in nature [5], is the available evidence sufficient to convince skeptics that stressful events are risk factors for the onset, recurrence, or exacerbation of skin disorders?

First, we identified differentially expressed genes from the two data sets and performed functional enrichment analysis. The psoriasis data set is involved in epidermis and skin development, and keratinocyte and epidermal cell differentiation, which regulate autophagy, T-cell proliferation, cytotoxicity, and T-cell communication, and negatively regulates T-cell maturation and differentiation. Related pathways involved in activated immune recognition include PRRs (pattern recognition receptors) signaling pathways such as NOD (nucleotide oligomerization domain)-like/RIG-I (retinoic acid-inducible gene I)-like receptor signaling pathway. Negatively regulates the WNT signaling pathway.

The anxiety disorder data set is involved in biological functions such as positive regulation of keratinocyte proliferation, regulation of receptor signaling pathway via JAK-STAT, regulation of lymphocyte-mediated immunity receptor signaling pathway via STAT, and lymphocyte-mediated immunity. GSEA analysis shows that it is involved in autophagy regulation, macroautophagy, activation of T-cell cytokines, negative regulation of T-cell proliferation, and mast cell migration, and negatively regulates T-cell selection and T-cell signal reception pathways and T-cell constant, differentiation, activation, and apoptosis processes.

In this study, we then used machine learning algorithms to screen genes to explore potential links between psychological stress and skin disease. The application of machine learning in genetic screening is a compelling new trend in today’s biomedical research. Machine learning algorithms can process large amounts of genetic data and learn hidden patterns and correlations. This allows us to more fully understand the interactions between genes and uncover potential biological implications. In this study, we combined the DEGs of two diseases with autophagy genes to generate 16 intersection genes. Used five machine learning algorithms to screen hub genes, and successfully screen out genes related to psychological stress and skin disease. These key genes were used to construct an ROC curve and verify its diagnostic efficacy. It can demonstrate good diagnostic efficiency on both the training set and the test set of psoriasis. This led to an in-depth study of the mechanisms of these genes in immune regulation and inflammatory responses.

Then we divided the disease into three subtypes based on these four hub genes and performed GSEA enrichment analysis. Disease subtype 1 activates the PRR-related signaling pathways and negatively regulates the development and differentiation of epidermal cells, keratinization, and cornification. It may indicate the overexpression of immune recognition, and reversely regulate normal development and differentiation of skin. Disease subtype 2 reversely expresses the PRR pathway and negatively regulates multiple autophagy-related biological processes, including mitochondrial autophagy, CMA (Chaperon-mediated autophagy), macroautophagy, etc. This disease subtype is shown to inhibit cellular self-cleaning mechanisms. Disease subtype 3 reversely expresses the PRR signaling pathway and is related to the positive regulation of epidermal cell differentiation and development, keratinization, cornification, and biological processes of skin development. GSEA analysis showed that the three disease subtypes were indeed enriched in different pathways and biological processes. Each has different characteristics, and it can be inferred that the prognosis of each disease is different. Molecular typing is helpful to determine its biological characteristics and prognosis, and to guide its clinical diagnosis and treatment. Treatment strategies for different subtypes reflect individualized treatment guided by molecular classification.

The immune infiltration analysis of the three disease subtypes also has different significance, and the expression of key genes is also different in the three disease subtypes. YLPM1 and SLC7A5 are significantly highly expressed in disease subtype 1. CASP7 and ARG1 are highly expressed in disease subtype 2. The immune correlation score of the four Hub genes shows that the CASP7 gene is positively correlated with gamma delta T cells (γδ T), and the ARG1 gene is highly negatively correlated with most immune cells. The SLC7A5 gene is positively related to Th17 cells.

Tregs are involved in autoimmune and allergic reactions and suppress autoreactive T cells. Tregs can specifically target self-antigens as well as harmful environmental antigens [17]. TIGIT (T-cell immunoglobulin and ITIM domain) is a co-inhibitory receptor of the immunoglobulin superfamily. It is expressed on multiple activated T-cell subsets, including CD4+ and CD8+ T cells, Tregs, and NK T cells. However, TIGIT is not detectable in naive T cells.

In recent years, studies have found that patients with psoriasis lack or downregulate TIGIT+ T cells, and the expression of TIGIT is negatively correlated with the severity of psoriasis [18]. In our findings, Tregs were consistently significantly downregulated in the three disease subtypes analyzed by GSEA. In single-cell analysis, Tregs and NK cells were relatively reduced compared to the control group, while the proportion of naive T cells increased significantly in the psoriasis group. Naive T cells are undifferentiated mature immune cells. When PRRs activate the NF-kb and JAK-STAT signaling pathways to activate CD4+ T cells, stimulating keratinocytes to secrete pro-inflammatory cytokines, naive T cells will differentiate into Th1, Th17, and Th22 (T helper 22). This means that the disease group lacks the mechanism to suppress the inflammatory response, and stimulates the inflammatory response.

Finally, in the single-cell sequencing analysis, there was a significant difference in the cell ratio between the disease group and the normal group. DC cells, T/NK cells, and monocytes/macrophages were significantly increased in the disease group, and keratinocyte was significantly decreased in the disease group. Both t-SNE and UMAP can ideally display the compositional differences of grouped cells, indicating that there is a significant difference in the cell composition of the disease group and normal group samples. The four genes in this study all have significantly high expression scores in T/NK cells and monocytes/macrophages in the disease group, indicating that the genes act on T/NK cells and monocytes/macrophages. Because the proportion of cells composed of T cells in the disease group increased significantly and had a significant high gene expression score, T cells were used for subsequent analysis in the single-cell subpopulation analysis.

T-cell subtype analysis showed that there was a significant difference in the proportion of T cells between the disease group and the normal group samples. CD4 memory T cells/NK cells decreased in the disease group, naive T cells/CD8 T cells increased significantly in the disease group, and there was no significant difference in the proportion of Treg cells between the two groups. In single-cell pseudotime analysis, T cells from the disease population are used to study the cell development process. The cell development heat map shows that the CASP7 gene is highly expressed in the early stages of T-cell development. This is consistent with the result that the gene immune correlation score CASP7 gene is positively correlated with γδ T cell.

γδ T cells are T cells with unique receptors. Their number is smaller than that of ordinary T cells, but they are particularly distributed in mucous membranes and epithelia. γδ T cells secrete IL-17/INF-r (Interferon-gamma)/IL-22 (Interleukin-22), forming a link between innate immunity and adaptive immunity. There have been many studies linking immune dysregulation to T-cell activity and psoriasis, where inflammation is an impairment of the IL-23 (Interleukin-23)/IL-17 axis. While Th17 cells and associated molecules, such as IL-17A, IL-17F, IL-22, and tumor necrosis factor (TNF-a), are known to be elevated in serum and psoriatic skin lesions, recent research suggests that Th17 cells are not the primary source of these pathogenic cytokines in psoriasis. [19]. In contrast, γδ T cells have been identified as the producers of IL-17A, IL-17F, and IL-22. [19]. Endogenous IL-1b is required for IL-17 production by dermal γδ T cells. Mechanistically, IL-1b activates the mTOR signaling pathway through IL-1R (interleukin-1 receptor)-MyD88 (Myeloid differentiation primary response gene 88), and the mTOR signaling pathway is related to the inhibition of the autophagy mechanism [8].

The SLC7A5 gene is involved in producing proteins that transport amino acids across cell membranes, with special emphasis on the mTOR signaling pathway. mTOR is a central regulator of cell growth, protein synthesis, and metabolism. Activation of the mTOR pathway inhibits autophagy, further triggering abnormal inflammatory responses. Our study shows that the SLC7A5 gene is highly expressed in the early stages of T-cell development, and the expression level decreases over time, but is highly expressed again in the late stages of cell development. It can be inferred that the mTOR signaling pathway affects the role of autophagy in psoriasis.

Cell communication analysis extracts disease group data and uses the AddModuleScore function to extract four hub genes for gene expression scoring. It can be seen that the hub gene is highly expressed in keratinocytes, DC cells, and T/NK cells. T cells were extracted separately and divided into two groups (“Psoriasis_UP_T” and “Psoriasis_DOWN_T”), according to the median of high and low gene expression groups for cell communication analysis. It can be seen that keratinocytes send ANGPTL signals to DC cells. DC cells send CD70, CXCL, GALECTIN, and MIF signaling pathways to other cells. The T-cell gene high-expression group only received the MIF/GALECTIN signaling pathway. The T-cell gene low-expression group received CD70, CXCL, GALECTIN, and MIF signaling pathway signals.

Compared with the high-expression group, the low-expression group received more signal pathways, showing that T cells in the low-expression group were more sensitive to multiple immune regulatory signals, which may mean that their immune regulatory system is more active. And it can make a relatively comprehensive response to a variety of immune stimuli in the surrounding environment. To some extent, this can be interpreted as a normal immune response, as the immune system needs to be able to respond to a variety of foreign pathogens and foreign bodies and maintain immune system balance and function. T cells with high expression may only be more sensitive to the MIF and GALECTIN signaling pathways, while being less responsive to signaling pathways such as CD70 and CXCL. Based on the above observations, it can be speculated that T cells with high expression of hub genes may be less reactive to some immune regulatory signals, leading to immune dysfunction and increased inflammatory response. However, this is only a preliminary hypothesis, and further experiments and analyses are needed to verify it.

## 4. Materials and Methods

### 4.1. Data Acquisition and Processing

The GSE13355 and GSE98793 data sets were obtained from the GEO database. Both data sets utilized the Affymetrix Human Genome U133 Plus 2.0 Array platform. The gene probes in these data sets were annotated using gene symbols, with the removal of probes lacking matches. Any gene probes with multiple matches were retained for further analysis.

The GSE13355 data set consisted of 58 samples of skin tissue obtained from individuals with psoriasis, while 64 samples of skin tissue from healthy controls were also included. On the other hand, GSE98793 focused on whole blood samples, comprising 64 samples from healthy controls and 128 samples from patients with Major Depressive Disorder. Among the MDD patients, there were subgroups with and without generalized anxiety disorder, each consisting of 64 patients. The analysis specifically targeted MDD patients with anxiety disorders.

These data sets offer a valuable resource for studying gene expression patterns linked to psoriasis and MDD, which are combined with anxiety disorders. The inclusion of healthy controls in both data sets allows for a comparative analysis to identify deferentially expressed genes associated with these conditions.

**Figure 16:**
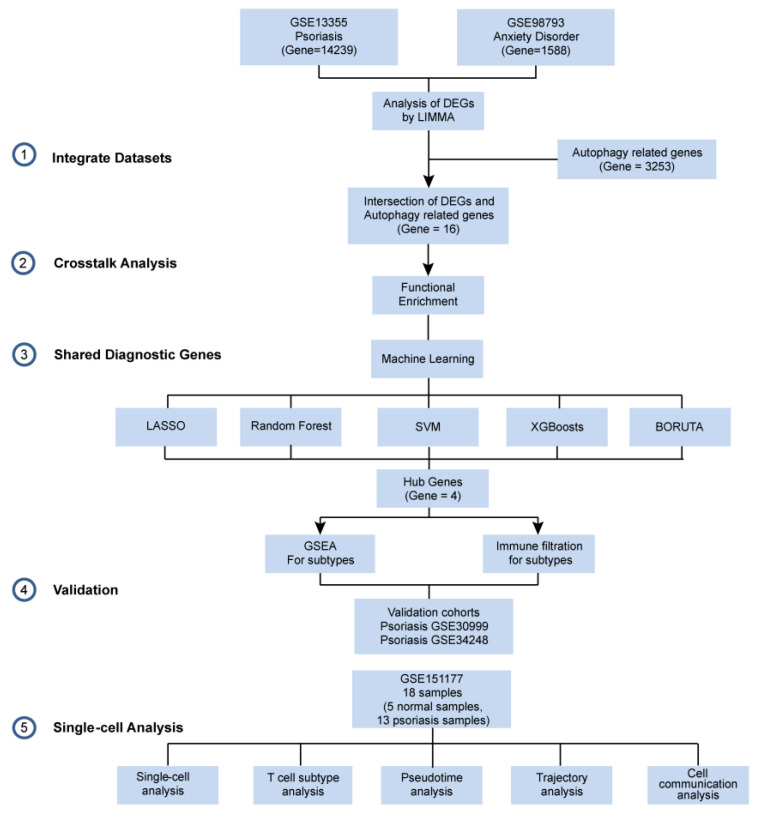
Workflow of the study.

We obtained two additional data sets, GSE30999 and GSE34248, from the GEO database as validation data sets. These data sets were also generated using the Affymetrix Human Genome U133 Plus 2.0 Array platform and consisted of skin biopsy samples. In both cases, one data set included samples from skin lesions, while the other included samples from non-lesion skin within the same general body region. In GSE30999, there were a total of 170 skin tissue samples from patients with psoriasis, evenly split between 85 non-lesion skin samples and 85 lesion skin samples. In GSE34248, there were a total of 28 skin tissue samples from psoriatic patients, with 14 samples from non-lesion skin and 14 samples from lesion skin. The workflow is shown in Figure 16.

### 4.2. Screening for Autophagy-Related Genes

The process began by querying the GeneCards database [20] with the keyword “autophagy”, resulting in an initial collection of 2969 ATGs. Subsequently, an additional set of 795 ATGs was incorporated from the HAMDb (Human Autophagy Moderator Database) database [21], and 222 genes were sourced from the HADb (Human Autophagy-dedicated Database) database [22]. To ensure uniqueness, any overlapping entries were eliminated through a refinement process. Ultimately, a comprehensive list of 3254 autophagy-related genes was identified.

### 4.3. Identification of Differentially Expressed Genes (DEGs)

To identify DEGs between healthy and diseased groups, the R package- LIMMA was utilized. Differential analysis of the processed gene expression matrix was performed using the LIMMA R suite [23]. The analysis was conducted on the processed gene expression matrix. The specific criteria were as follows: The cutoff criteria for the psoriasis data sets in GSE13355 included adjusted *p*-value < 0.05 and |log fold change (FC)| > 0.5. Similarly, the threshold requirements for the anxiety disorder data sets in GSE98793 also included adjusted *p*-value < 0.05 and |log fold change (FC)| > 0.

Utilized the ggplots suite for visualization of both data sets. Generated a heatmap and volcano diagram to visually represent the differentially expressed genes (Figure 1). This approach allowed for the identification and visualization of genes that exhibited significant expression differences between lesional and healthy samples in both the psoriasis and anxiety disorder data sets.

### 4.4. Identification of the Shared DEGs of Autophagy

We conducted an intersection analysis between the DEGs from GSE13355 and GSE98793, revealing a shared set of 44 DEGs. We then further intersected this set of 44 DEGs with the 3253 ATGs, resulting in a total of 16 Autophagy-Related DEGs (Figure 2).

### 4.5. Functional Enrichment Analysis

To elucidate the biological significance of the genes and their functions, differentially expressed genes were analyzed using the R package “clusterProfiler” for Gene Ontology (GO) and Kyoto Encyclopedia of Genes and Genomes (KEGG) pathways [24]. Our significance level was set at a *p*-value threshold of less than 0.05.

In GO analysis, the identified DEGs were divided into three main categories: molecular function (MF), biological process (BP), and cellular composition (CC). Subsequently, we conducted a KEGG enrichment analysis to predict the specific signaling pathways in which these DEGs may be involved. For a term to be considered significant, it had to meet the criteria of having a *p*-value below 0.05 and a q-value (false discovery rate, FDR) below 0.25.

### 4.6. Gene Set Enrichment Analysis (GSEA)

In order to gain deeper insights into the biological mechanisms underlying the association between psoriasis and anxiety disorder, we conducted Gene Set Enrichment Analysis (GSEA) to identify potential molecular pathways involved [25,26]. For this analysis, we employed the “c2.cp.kegg.v7.0.entrez.gmt” reference gene set, which was obtained from the Molecular Signature Database (MSigDB) [27]. Our significance level was set at an FDR level less than 0.25, and *p*-value less than 0.05.

### 4.7. Using Machine Learning Algorithms to Filter Core Hub Regulators

Based on the 16 Autophagy-Related DEGs, LASSO regression [28], SVM (support vector machine) [29], RF (Random Forest) [30], XGBoost (eXtreme Gradient Boosting) [31], and BORUTA algorithm [32] were employed to filter important diagnostic variables.

The analysis involved determining the intersection of the signature genes identified by the five algorithms. ROC curves were then constructed one by one to assess the predictive ability of the feature genes in both the training and validation sets [33]. The following steps were taken:

First, Intersection of Signature Genes [34]. Signature genes were identified by five different algorithms. The intersection of these genes across algorithms was determined. Next is ROC Curve Analysis. ROC curves were generated for these overlapping genes. The R package-pROC was employed to calculate the AUC values, which assessed the predictive performance of the genes [35]. Then, the predictive ability of the four hub genes was verified in the validation data sets. This comprehensive analysis aimed to evaluate the consistency and predictive ability of the identified signature genes across different algorithms and in both the training and validation data sets.

### 4.8. Consensus Clustering

Utilizing the hub genes to perform unsupervised clustering on the psoriasis gene data set using the “ConsensusClusterPlus” method [36]. The optimal number of subtypes (denoted as k) was determined by examining key metrics including CDF curves, consensus scores, and consensus matrices. These analyses help define distinct subtypes within the psoriasis patient population.

### 4.9. Differences and Correlation Analysis of Immune Characteristics

The CIBERSORT algorithm was used to figure out the relative abundance and immunoreactivity of 22 immune cells (LM22) [37,38,39]. Utilizing the LM22 gene set, samples with statistically insignificant results (*p*-value > 0.05) were excluded. The Wilcoxon test was then utilized to compare the differences in the proportions of the 22 immune cells among the three disease subtypes. ssGSEA also investigated the status of immune cells and the hub genes. The relationship between the five hub genes and the immune cells and immune response activity was determined using Spearman’s rank correlation analysis.

### 4.10. Transcription Factor (TF)–Gene Interactions

Transcription factors are proteins involved in the process of converting, or transcribing, DNA into RNA. These proteins can regulate the transcription of their genes, thereby controlling the activation or repression of target genes. In this study, we employed NetworkAnalyst 3.0 (https://www.networkanalyst.ca/NetworkAnalyst/home.xhtml, accessed on 20 September 2023) to investigate the interplay between shared genes and transcription factors. This analysis aimed to evaluate which transcription factors influence gene expression and functional pathways.

Transcription factor targets derived from the JASPAR TF binding site profile database. Subsequently, a TF–gene regulatory network was established and visually represented using Cytoscape.

### 4.11. Statistical Analysis

The analysis of the data was conducted using R software (version 4.3.0.1). Group comparisons were carried out using the Wilcoxon test, with significance set at *p* < 0.05 to determine statistically significant differences.

### 4.12. Single-Cell RNA-Sequencing Data

We utilized the Seurat package (v5.0.1) for comprehensive data processing, dimension reduction, and cell clustering [40]. The single-cell gene expression profile was filtered to exclude cells with either over 25% mitochondrial genes or fewer than 200 cells. Principal Component Analysis (PCA)-based linear dimensional reduction and clustering visualization were conducted through the RunPCA and RunTSNE functions in Seurat, respectively. Manual annotation was carried out for cell identification [41,42].

Pseudotime and cell trajectory analysis of single cells were performed using the Monocle 2 package (version 20) [43]. The resulting cellular trajectory, ordered in pseudotime with two branches, was visualized, and genes along the trajectory were subjected to subsequent analysis.

For cellular interaction network analysis, CellChat was applied to our scRNA-seq data set [44]. This allowed us to infer the intercellular communication status of immune cells and predict potential signaling pathways altered by hub genes in psoriasis.

## 5. Conclusions

In summary, this study used machine learning algorithms to identify four key hub genes as diagnostic genes. Enrichment analysis showed that these genes are indeed related to T cells, autophagy, and immune regulation, and have good diagnostic efficacy. Single-cell analysis provides insights into the pathogenesis of psoriasis, expanding our understanding of key cellular players, including inflamed keratinocytes and their interactions with immune cells. We found that anxiety disorders are related to autophagy regulation, immune dysregulation, and increased inflammatory response, and are reflected in the onset and exacerbation of skin inflammation. The psoriasis CASP7 gene is involved in the development and differentiation of T cells, as well as the process of immune signaling and immune regulation, and deserves further study. In addition, potential biomarkers common between psoriasis and anxiety disorders were identified, which are expected to aid in the prediction of disease diagnosis and the development of personalized treatments.

## Data Availability

In this study, the public data sets were downloaded and analyzed, and can be found in the GEO data repository and include the accession numbers as follows: GSE13355, GSE98793, GSE30999, GSE34248, and GSE151177.
